# Amoxicillin-induced aseptic meningitis: clinical features, diagnosis and management

**DOI:** 10.1186/s40001-023-01251-y

**Published:** 2023-08-27

**Authors:** Zhiqiang Fan, Yang He, Wei Sun, Zuojun Li, Chao Ye, Chunjiang Wang

**Affiliations:** 1 Department of Pharmacy, The First Hospital of Hunan University of Chinese Medicine, Changsha, 410007 Hunan China; 2 College of Pharmacy, Changsha Medical University, Changsha, 410219 Hunan China; 3 Department of Pharmacy, The Third Xiangya Hospital, Central South University, No. 138 Tongzipo Road, YueLu District, Changsha, 410013 Hunan China; 4 Department of Pharmacy, The Third Hospital of Changsha, Changsha, 410015 Hunan China

**Keywords:** Aseptic meningitis, Drug-induced aseptic meningitis, Amoxicillin, Amoxicillin-clavulanate, Pharmacovigilance

## Abstract

**Objectives:**

The clinical features of aseptic meningitis associated with amoxicillin are unknown. The main objective of this study was to investigate the clinical characteristics of amoxicillin-induced aseptic meningitis (AIAM) and provide a reference for clinical diagnosis and treatment.

**Methods:**

AIAM-related studies were collected by searching the relevant databases from inception to October 31, 2022.

**Results:**

AIAM usually occurred 3 h to 7 days after amoxicillin administration in 13 males and 9 females. Twenty-one patients (95.5%) had recurrent AIAM with a total of 62 episodes. Fever (19 cases, 86.4%) and headache (18 cases, 81.8%) were the most common symptoms. Typical cerebrospinal fluid (CSF) findings were leukocytosis (100%) with lymphocytic predominance (14 cases, 63.6%), elevated protein (20 cases, 90.1%), normal glucose (21 cases, 95.5%) and negative culture (21 cases, 100%). Brain magnetic resonance imaging showed mild meningeal enhancement in one patient. The symptoms resolved mainly within 1–4 days after drug discontinuation in all patients.

**Conclusion:**

Clinical attention should be given to the adverse effects of AIAM. The medication history of patients with suspected meningitis should be investigated to avoid unnecessary examination and antibiotic treatment.

## Introduction

Meningitis is characterized by diffuse inflammatory changes in the pia mater caused by various biological pathogenic factors, such as bacteria, viruses, fungi or protozoa, tumors, cancers, and autoimmune systemic diseases invading the pia mater and spinal cord membrane [1–3]. Aseptic meningitis (AM) is defined as the absence of evidence of pathogen infection but the presence of associated neurological symptoms and cytological abnormalities in the cerebrospinal fluid (CSF) [4]. Drugs are an important factor in AM, including nonsteroidal anti-inflammatory drugs (NSAIDs), antibiotics, intravenous immunoglobulin, antiepileptic drugs, and monoclonal antibodies [5].

The incidence of drug-induced aseptic meningitis (DIAM) remains unreported. Antibiotic-associated DIAM is most often caused by trimethoprim with or without sulfamethoxazole and penicillin-derived antibiotics [5, 6]. Analysis of 329 cases of drug-induced aseptic meningitis showed that 11% of the drugs were antibiotics, of which amoxicillin accounted for 5% [7]. The incidence, pathogenesis, treatment and prevalence of amoxicillin-induced aseptic meningitis (AIAM) are not widely reported. Knowledge of AIAM is limited to individual reports. AIAM has not been labeled as a potential adverse effect in most countries. Assessment of AIAM is still variable today and is a diagnostic and management dilemma for clinicians. Here, we collected case reports of AIAM, explored its clinical features, and provided a reference for clinical diagnosis and treatment.

## Methods

### Retrieval strategy

We collected case reports, case series, clinical studies and reviews of amoxicillin-induced aseptic meningitis by searching Chinese databases (Wanfang, China VIP, CNKI) and English databases (PubMed, Embase, Cochrane) from establishment to October 31, 2022. The searches were performed using subject and free words, including “amoxicillin” [MeSH] OR “amoxicillin-clavulanate” [MeSH] OR “antibiotics” [MeSH] OR “β-lactam antibiotics” OR “drug” AND “meningitis” [MeSH] OR “aseptic meningitis” [MeSH] OR “drug-induced aseptic meningitis” [MeSH] AND “headache” [MeSH] AND “pharmacovigilance” [MeSH].

### Inclusion and exclusion criteria

Case reports and clinical study series were included. Reviews, basic studies, replication studies, and studies of other antibiotic-induced AM were excluded.

### AIAM definition

AIAM was defined as the time relationship with amoxicillin-containing products, CSF leukocytosis (> 5 cells/µl), negative culture, and symptom recovery after drug withdrawal. For patients with several episodes, we selected the most recent episode for data extraction.

### Data Extraction

Two independent authors collected patients' nationality, sex, age, drug usage and dosage, meningitis symptoms, neuroimaging results, cerebrospinal fluid results, treatment regimens and clinical outcomes and then summarized the data. For patients with several episodes, we selected the most recent episode for data extraction.

## Results

### Basic data

**Fig. 1:**
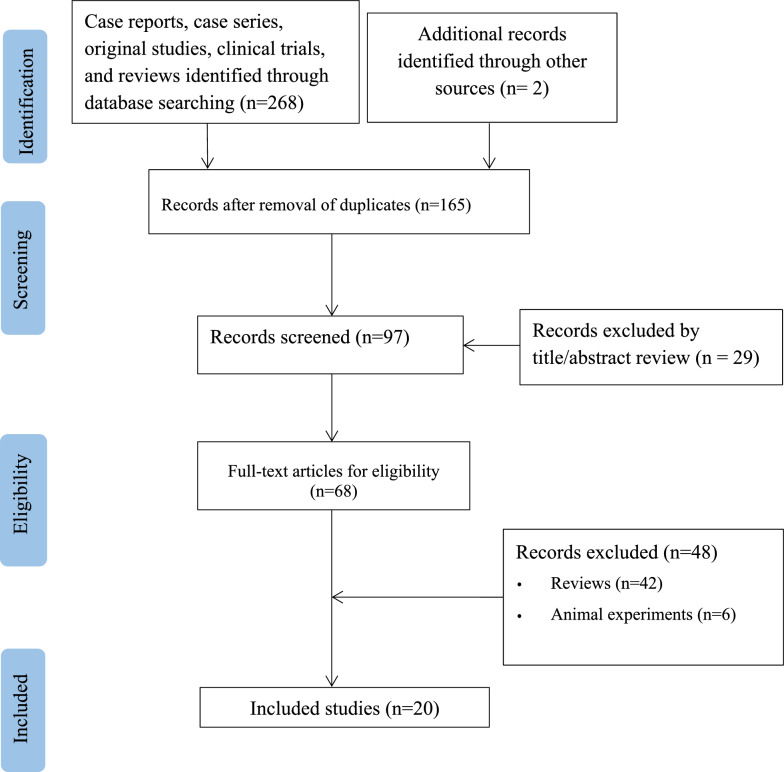
Flowchart of the study selection process for reported cases of amoxicillin-induced aseptic meningitis

The literature screening process is shown in Fig. 1. A total of 22 patients with AIAM from 20 articles were included [8–27]. The basic information of the 22 patients is summarized in Table 1. The median age of 9 females and 13 males was 62.5 years (range 30–86), and the patients were mainly from Europe (14 cases, 63.6%), North America (6 cases, 27.3%), and Asia (2 cases, 9.1%). Of the 22 patients, 13 (59.1%) received amoxicillin, and 9 (40.9%) received amoxicillin clavulanate. The onset time of symptoms varied from 3 h to 7 d after administration. Six patients (27.3%) exhibited symptoms within 12 h after administration, 10 patients (45.5%) after 1 to 3 days, and 5 patients (22.7%) after 4 to 7 days.

**Table 1:** Basic information of the 22 patients included

References	Country	Age/sex	Medical history	Indication	Type	Dose	Route of administration	Latency period	No. of episodes
[8]	Portugal	63/m	na	Dental procedure	A	na	Oral	3 d	2
[9]	Croatia	30/f	na	Genital infection	AC	na	Oral	2 d	2
[10]	Canada	73/m	COPD, CKD	Otitis media	A	500 mg BID	Oral	2 d	2
[11]	France	78/m	na	Broncho pneumopathy	A	na	na	3 d	3
[12]	France	86/m	na	Skin infection	AC	1 g BID	Intravenous	2 d	2
[13]	Switzerland	76/f	na	Skin infection	AC	na	na	3 d	3
[14]	Spain	40/f	Hypothyroidism	Dental pain	AC	na	na	4 d	2
[15]	Germany	62/m	na	Dental procedure	AC	500 mg/125 mg	Oral	6 h	2
[16]	USA	55/m	na	Dental procedure	A	500 mg	Oral	12 h	2
[17]	Israel	82/f	na	Pneumonia	A	1.5 g daily	Oral	3 d	7
[18]	Israel	77/m	na	Dental troubles	A	500 mg daily	Oral	7 d	3
[19]	USA	58/f	Multiple food allergy	Broncho pneumopathy	A	na	na	3 h	2
[20]	USA	58/f	Multiple food allergy	Dental procedure	A	2 g	na	4 h	2
[21]	USA	72/m	na	Sore throat	A	500 mg tid	Oral	4 d	4
[22]	USA	80/f	na	Surgical procedure	A	500 mg	Oral	4 d	1
[23]	Spain	62/m	na	Dental pain	AC	na	Oral	4 d	4
[23]	Spain	58/m	na	Whitlow	AC	na	Oral	na	3
[24]	Spain	71/m	Hypertension, prostate cancer, hyperuricemia	Dental procedure	A	na	na	2 d	2
[25]	Spain	66/m	na	Dental pain	A	na	Oral	2 d	4
[26]	Spain	58/m	na	skin infection	AC	na	Oral	1 d	3
[27]	France	62/f	na	Dental troubles	A	500 mg	Oral	0.5 d	4
[27]	France	32/f	na	Dental troubles	AC	na	Oral	0.5 d	4

*AC* Amoxicillin clavulanic acid, *A* amoxicillin, *COPD* Chronic obstructive pulmonary disorder, *CKD* chronic kidney disease, *na* not available

Two patients (9.1%) had a history of multiple food allergies. Of the 16 patients (27.3%) for whom the administration method was described, 15 (68.2%) were administered orally, and 1 patient (4.5%) was administered by intravenous injection. Both amoxicillin and amoxicillin clavulanate doses were within the recommended range in the 10 patients (45.5%) with records.

### Clinical symptoms

The clinical data of 22 patients are shown in Table 2. Twenty-one patients (95.5%) had recurrent AIAM with a total of 62 episodes, with a maximum of 7 episodes in one patient (4.5%). Patients usually presented with fever (19 cases, 86.4%), headache (18 cases, 81.8%), nuchal rigidity (7 cases, 31.8%), photophobia (6 cases, 27.3%) and nausea (6 cases, 27.3%). Headache was severe and progressive, with two patients (9.1%) presenting with pressure-like global headache and one patient presenting with occipital pain. Other symptoms included vomiting (4 cases, 18.2%), myalgia (4 cases, 18.2%), chills (3 cases, 13.6%), and phonophobia (2 cases, 9.1%). Rare complications included behavioral confusion, aphasia, apraxia, coma, lethargy, difficulty walking, weakness, and diarrhea.

**Table 2:** Clinical manifestations, cerebrospinal fluid examination results, treatment and prognosis of the 22 included patients

References	Clinical symptoms	CSF cytology	CSF protein (mg/dl)	CSF glucose (mg/dl)	CSF cultures	Treatment	Time to symptom resolution
[8]	Fever, headache	Pleocytosis 25/μL (lymphocytic predominance)	100	54	Negative	D, recovery	4 d
[9]	Fever, headache, nuchal rigidity, myalgia, phonophobia, photophobia	Lymphocytes 87%	Elevated	Normal	Negative	D, AET, recovery	3 d
[10]	Fever, headache, confusion, nausea	Leukocytosis 31/μL (87% lymphocytes)	57.2	Normal	Negative	D, AET, recovery	3 d
[11]	Fever, confusion	Pleocytosis	Elevated	Normal	Negative	D, recovery	A few days
[12]	Fever, confusion, coma, nuchal rigidity	Leukocytes 18/μL	60	Normal	Negative	D, recovery	4 d
[13]	Fever, headache, nuchal rigidity	Pleocytosis 63/μL (monocytes 62/μL)	47	Normal	Negative	D, recovery	NA
[14]	Fever, headache, nuchal rigidity	Lymphocyte 79/μL	78	77	Negative	D, AET, recovery	1 d
[15]	Fever, headache	Leucocyte 54/μL (82% lymphocytes, 12% monocytes, 4% lymphoid cells, 2% granulocytes)	94	62	Negative	D, recovery	7 d
[16]	Fever, headache, chills, phonophobia	Leucocyte 70/μL (100% lymphocytes)	61.2	51	Negative	D, recovery	3 d
[17]	Fever, headache, myalgia, confusion, nuchal rigidity	Lymphocytes 640/μL	1380	55.8	Negative	D, AET, recovery	4 d
[18]	Fever, headache, chills, nuchal rigidity	Pleocytosis 20/μL (80% mononuclear)	91	Normal	Negative	D, AET, recovery	2 d
[19]	Fever, headache, photophobia, nausea, vomiting, chills, myalgias	Neutrophils 92%	Elevated	Normal	Negative	D, recovery	12 h
[20]	Fever, headache, nausea, vomiting	Pleocytosis 624/μL (90% neutrophils and 4% lymphocytes), RBC 17/μL	228	Normal	Negative	D, AET, recovery	2 d
[21]	Photophobia, mental status changes, photophobia, lethargic and inattentive	Leukocytosis 84/μL (79% lymphocytes), RBC 20/μL	97	76	Negative	D, AET, recovery	14 d
[21]	Fever, headache, nausea, vomiting	Leukocytosis 30/μL(66% monocytes, 21% lymphocytes, 13% neutrophils)	70	73	Negative	D, AET, recovery	9 d
[22]	Fever, headache	Leukocytosis 44/μL (20% polymorphonucleocytes; 80% mononuclear), RBC 70/μL	80	50	Negative	D, recovery	4 d
[23]	Fever, headache	Leukocytosis 130/μL (100% lymphocytes), RBC 70/μL	86	48	Negative	D, recovery	na
[24]	Headache, photophobia, nausea dysbasia	Leukocytosis 46/μL (85% neutrophils)	109	57	Negative	D, AET, recovery	3 d
[25]	Fever, headache	Lymphocytic pleocytosis	na	na	Negative	D, symptomatic treatment, recovery	4 d
[26]	Fever, headache, photophobia	Pleocytosis 130/μL (90% lymphocytes)	86	89	Negative	D, recovery	na
[27]	Fever, headache, nausea, vomiting	Lymphocytic pleocytosis	Normal	Normal	Negative	D	na
[27]	Headache, nausea, photophobia, myalgia, asthenia, nuchal rigidity	Lymphocytic pleocytosis	Elevated	Normal	Negative	D	na

*AET* Antibiotic experiential treatment, *CSF* cerebrospinal fluid, *D* Discounted, *CT* Computed tomography, *MRI* Magnetic resonance imaging, *na* not available, *RBC* Red blood cells

### Laboratory tests and brain imaging

CSF examination of 21 patients found that glucose was normal (21 cases, 100%); protein was elevated in 20 cases (95.2%) and normal in 1 case (4.8%). The median value of protein was 91 mg/dL (range: 41–2560). Cytological analysis of CSF revealed leukocytosis in 22 patients, including 14 patients (63.6%) with lymphocytosis, 4 patients (18.2%) with monocytosis, and 3 patients (13.6%) with neutrophilia. Red blood cells were found in 3 patients (13.6%).

Among the 22 patients, 18 patients underwent magnetic resonance imaging (MRI) examination; 17 patients showed no abnormality, and 1 patient showed mild meningeal enhancement on MRI. Computed tomography (CT) was performed in 17 patients with no abnormalities. CSF cultures found no evidence of bacteria or viruses in 22 patients.

### Treatment and prognosis

Amoxicillin and amoxicillin clavulanate were eventually discontinued in all patients. Ten patients (45.5%) received empirical antibiotic treatment. Meningitis symptoms recovered in 20 patients (90.9%) within 12h-14d after drug withdrawal, and the clinical outcome was not described for 2 patients (9.1%).

## Discussion

DIAM is a rare entity, and the onset time is variable, ranging from a few minutes to several months [28, 29]. Moreover, clinical signs and cerebrospinal fluid examination results vary greatly. The clinical manifestations are consistent with meningoencephalitis, including fever, headache, photophobia, neck stiffness, nausea, vomiting, and myalgia [30]. AIAM appeared at 3 h ~ 7 d after administration. DIAM may initially be confused with viral or bacterial meningitis. If other inducements are excluded, the diagnosis of DIAM is based on the time between administration and symptom onset and the rapid disappearance of symptoms after drug withdrawal. Several pathophysiological factors may contribute to DIAM, such as autoimmune disease, migraine, immune dysregulation and genetic predisposition [31]. Meningitis was caused by intravenous penicillin in one woman [32]. Cephalosporins, including cefazolin, ceftriaxone and ceftazidime, are also associated with aseptic meningitis. [7] One woman developed several episodes of aseptic meningitis due to exposure to cefalexin, cefazolin, and ceftazidime [33]. This patient should avoid cephalosporins and consider herself cross-allergic to them. It is not known whether AIAM would show cross sensitivity with other penicillins, and we suggest that it would be advisable to avoid the use of other penicillins in patients who have been diagnosed with AIAM.

The pathophysiology of DIAM is unclear, and two main mechanisms may be involved. One is the direct toxicity of drugs, and the other is related to immune hypersensitivity [30]. The current study suggests that AIAM may be a delayed type 4 hypersensitivity reaction or T-cell-mediated hypersensitivity reaction. Laboratory results in one AIAM patient did not support type I or type III reactions, and no specific IgE or C1q were detected [15]. T-cell activation was detected in another 2 AIAM patients [27]. The occurrence of AIAM independent of amoxicillin dose may indicate that this is an allergic reaction. Men are at higher risk for AIAM than women. A possible explanation is that the immune response differs by sex. [34]

In our study, we found that fever and headache were the most common complaints. Five patients developed symptoms of meningoencephalitis, characterized by neurological symptoms such as disturbances of consciousness and coma. CSF examination results vary in different DIAM cases, usually in terms of CSF cytosis, which is mainly neutrophilic, but some patients have CSF lymphocytosis and eosinophilia with unchanged leucocytes. Protein levels in the CSF are usually high, while glucose levels are normal, although some cases are described as having reduced glucose levels [35]. The typical CSF manifestation of AIAM is neutrophilic lymphocytosis, and the number of white blood cells is tens to hundreds per microliter, with normal glucose levels and variably elevated protein levels. In general, clinical symptoms and cerebrospinal fluid findings are not helpful in distinguishing between drug-induced aseptic meningitis and bacterial meningitis. Blood tests and brain CT or MRI scans were not diagnostic. Determining the temporal relationship between amoxicillin use and aseptic meningitis is critical for diagnosis. Compared with bacterial meningitis patients, the CSF glucose level of AIAM patients is usually lower, while that of DIAM patients is normal [36]. Another difference between bacterial meningitis and DIAM is the high level of C-reactive protein (CRP). The symptoms of DIAM usually recover within 10–14 days, which is different from viral meningitis [18]. However, the symptoms of AIAM disappeared within 7 days.

The causative drug needs to be discontinued in cases of DIAM [30]. Symptoms associated with meningitis can be given symptomatic treatment, such as pain relievers for headache and antiemetic drugs for nausea and vomiting. Patients with immunoglobulin (IVIG)-related DIAM received hydration, analgesics, and systemic corticosteroids [37–39]. Antitumor necrosis factor drugs were also considered in some serious cases [40]. However, there was no significant difference in the clinical course, regardless of whether the patient received treatment.

AIAM is reversible and usually does not require any further treatment after drug withdrawal. When AIAM is suspected, the drug should be discontinued if possible. A third-generation cephalosporin may be given empirically to avoid any risk of infection until CSF results are available [31]. AIAM patients may relapse after challenge. We do not recommend that patients rechallenge amoxicillin or amoxicillin clavulanic acid in cases of previous aseptic meningitis. Rechallenging amoxicillin or amoxicillin clavulanate potassium can lead to a recurrence of aseptic meningitis.

### Limitations

There are some limitations to our study. First, the sample size was small, our study was limited to case series and single case reports, and statistical analysis was hampered, making it difficult to confirm or disprove certain clinical features associated with AIAM. Second, the level of detail provided was different in each case. Therefore, it is subject to a certain degree of data interpretation. Despite its limitations, we believe that this study still has some reference value.

## Conclusion

There is a possible association between amoxicillin-containing products and the risk of aseptic meningitis. Identifying AIAM is a challenge, and patients with meningitis should be asked about their medication history in detail to avoid unnecessary testing and treatment.

## Data Availability

The data used to support the findings of this study are included within the article.
